# Comparison of five video-assisted intubation devices by novice and expert laryngoscopists for use in the aeromedical evacuation environment

**DOI:** 10.1186/s40779-017-0129-2

**Published:** 2017-06-14

**Authors:** Matthew C. Wallace, SSgt Tyler Britton, Robbie Meek, Sharon Walsh-Hart, Col Todd E. Carter, Steven J. Lisco

**Affiliations:** 1 Department of Anesthesiology, University of Cincinnati Medical Center, 231 Albert Sabin Way, P.O. Box 670531, Cincinnati, OH 45267–0351 USA; 2 University of Cincinnati Medical Center, C-STARS Program, 234 Goodman Street, Cincinnati, OH 45202 USA; 3 UC Health Air Care and Mobile Care, 3200 Burnet Avenue, Cincinnati, OH 45229 USA; 4 Department of Anesthesiology, University of Nebraska Medical Center, 984455 Nebraska Medical Center, Omaha, NE 68198–4455 USA

**Keywords:** Simulation, Difficult airway, Novice, Expert, Military, Video, Laryngoscopy, Light emission

## Abstract

**Background:**

The critically ill or injured patient undergoing military medical evacuation may require emergent intubation. Intubation may be life-saving, but it carries risks. The novice or infrequent laryngoscopist has a distinct disadvantage because experience is critical for the rapid and safe establishment of a secured airway. This challenge is compounded by the austere environment of the back of an aircraft under blackout conditions. This study determined which of five different video-assisted intubation devices (VAIDs) was best suited for in-flight use by U.S. Air Force Critical Care Air Transport Teams by comparing time to successful intubation between novice and expert laryngoscopists under three conditions, Normal Airway Lights on (NAL), Difficult Airway Lights on (DAL) and Difficult Airway Blackout (DAB), using manikins on a standard military transport stanchion and the floor with a minimal amount of setup time and extraneous light emission.

**Methods:**

A convenience sample size of 40 participants (24 novices and 16 experts) attempted intubation with each of the 5 different video laryngoscopic devices on high-fidelity airway manikins. Time to tracheal intubation and number of optimization maneuvers used were recorded. Kruskal-Wallis testing determined significant differences between the VAIDs in time to intubation for each particular scenario. Devices with significant differences underwent pair-wise comparison testing using rank-sum analysis to further clarify the difference. Device assembly times, startup times and the amount of light emitted were recorded. Perceived ease of use was surveyed.

**Results:**

Novices were fastest with the Pentax AWS in all difficult airway scenarios. Experts recorded the shortest median times consistently using 3 of the 5 devices. The AWS was superior overall in 4 of the 6 scenarios tested. Experts and novices subjectively judged the GlideScope Ranger as easiest to use. The light emitted by all the devices was less than the USAF-issued headlamp.

**Conclusions:**

Novices intubated fastest with the Pentax AWS in all difficult airway scenarios. The GlideScope required the shortest setup time, and participants judged this device as the easiest to use. The GlideScope and AWS exhibited the two fastest total setup times. Both devices are suitable for in-flight use by infrequent and seasoned laryngoscopists.

## Background

Emergent intubation in the critically ill or injured patient undergoing military aeromedical evacuation (AE) may be a challenging but lifesaving intervention. Intubation carries risks, including difficulty visualizing the vocal cords and resultant inability to correctly place the endotracheal tube, damage to laryngeal structures, bleeding in the laryngopharynx, and inadvertent esophageal intubation [1]. The novice laryngoscopist is at a disadvantage because experience is critically important for rapid and successful intubation of the trachea, and airway management is as much an art as it is a science [2, 3]. The challenge of intubating the airway is compounded when environmental conditions are austere, such as in the back of an aircraft under blackout conditions in the military combat setting.

While the skill of tracheal intubation via direct laryngoscopy is taught to many healthcare professionals, it is a difficult skill to acquire and maintain [4]. Serious consequences may result from a poorly performed intubation attempt. The rate of airway-related complications correlates with an increased number of intubation attempts. The increased number of laryngoscopy attempts increases the incidence of hypoxemia, aspiration, bradycardia, and cardiac arrest [5]. Successful intubation occurring on the initial attempt is imperative. Several studies compared direct laryngoscopy using a Macintosh blade with video-assisted intubation devices (VAIDs) and found a more rapid acquisition of skills and faster and more consistent intubation in difficult scenarios and less theoretical dental trauma (in intubations performed on a manikin) [3, 6–9]. First-time users of video assisted intubation devices have an improved view of the glottis during difficult airway situations compared to direct laryngoscopy [10]. The skill of novice laryngoscopists diminishes rapidly over a period of several months without intervening practice [11].

Intubation of the normal airway in a well-lit environment may be challenging for the inexperienced or infrequent laryngoscopist. This challenge is multiplied in the AE environment where U.S. Air Force Critical Care Air Transport Teams (CCATTs) may need to intubate a patient’s airway under suboptimal conditions, such as low light, on upper level stanchions or the floor of the aircraft, or when providers cannot easily place themselves at the head of the patient due to space restrictions of the aircraft. The use of a VAID in this type of environment likely increases the first-pass success rate because it provides a clear visualization of the glottic opening for providers, who may or may not be seasoned or frequent laryngoscopists [8].

The Air Force Medical Evaluation Support Activity (AFMESA) at Fort Detrick, Maryland, published a market research report on various VAIDs (Video Assisted Intubation Devices Market Research Report, AFMESA-MR-09-304; distribution limited to Government agencies only) in June 2009. The market research team chose certain characteristics as critical in a VAID for in-flight use (Appendix 1). AFMESA identified nine commercially available devices that fit or closely approximated the requirements. The market research report reviewed many “on paper” capabilities of the VAID, but it did not field-test the dynamic properties of these devices that may lead to improved patient safety outcomes in the CCAT environment.

The present study used simulation to determine which of the top three VAIDs from the AFMESA list, plus two others in current, routine clinical use are best suited for the CCATTs environment in ease and rapidity of intubation by both novice and seasoned laryngoscopists with minimal extraneous light emission and setup time.

## Methods

The University of Cincinnati Medical Center’s (UCMC) Institutional Review Board (IRB) reviewed this study and classified it as exempt from full IRB review because it did not meet its requirements for research involving human subjects. The Air Force Research Laboratory IRB deferred to UCMC’s conclusion. Participants were classified based on previous experience with laryngoscopy and included residents in anesthesiology, emergency medicine, and surgery, student-registered nurse anesthetists, respiratory therapists, anesthesiology attending physicians, and emergency medicine and critical care nurses. The convenience sample included a Novice group (24 participants), who self-identified as having less than 30 lifetime intubations, and an Expert group (16 participants), which required a self-reported 30 or more lifetime intubations.

Each participant used each of the 5 devices (Table 1) in a randomized order by blindly choosing from identical cards that were pre-printed with each device name. No specific training was performed on the devices prior to use in the study because participants’ local purchasing authority may acquire different devices than what is issued as part of the CCATTs equipment set. Therefore, participants may use a different device at their home hospital than when deployed in a CCATTs capacity. Each participant was permitted up to three attempts with a maximum time allotment of 2 min per attempt to successfully intubate the manikin. Subjects had a total of 6 min per device to achieve tracheal intubation and the opportunity to reassess their technique and make a new attempt. The time to successful intubation was recorded.

**Table 1:** Tested video-assisted intubation devices (VAIDs)

Device name	Manufacturer	Manufacturer location
Airtraq	Prodol Meditec, S.A.	Vizcaya, Spain
AWS	Pentax Medical Company	Montvale, NJ, USA
C-MAC	Karl Storz Endoskope GmbH & Co, KG	Tuttlingen, Germany
Coopdech VLP-100	Daiken Medical Co, Ltd	Osaka, Japan
GlideScope Ranger	Verathon, Inc.	Bothell, WA, USA

Permitted optimization maneuvers were verbalized to each participant prior to their attempts at intubation and included external laryngeal manipulation (ELM) and simple manipulation of the manikin’s head. The number of optimization maneuvers used for each device was recorded as a count variable with integers starting at 0. All five devices were compared simultaneously using a chi-square test to determine whether there was any significant difference in the number of optimization maneuvers used for each device. Participants were given a brief survey after the use of each VAID consisting of a subjective “ease of use” evaluation using a 5-point Likert scale, ranging “5 = Extremely easy” to “1 = Extremely difficult”. The results of participants’ attempts were not communicated to their employers or instructors.

**Fig. 1:**
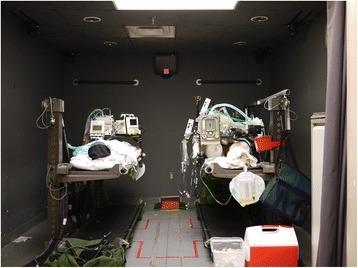
View of the simulation laboratory under full light conditions

**Fig. 2:**
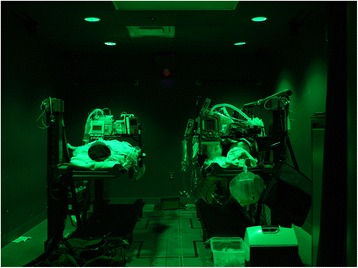
View of the simulation laboratory under simulated blackout conditions

The study was performed in a high-fidelity patient simulator lab, which is used for patient care simulation in the CCATT Advanced Training Course at UCMC. This area permitted use of the lab’s standard white room lights for the “lights-on” portions of the study (Fig. 1, View of the simulation laboratory under full light conditions) and the existing green ceiling lights for the “blackout” portions, which simulates the conditions in an aircraft during takeoffs, landings, and night missions in a combat zone (Fig. 2, View of the simulation laboratory under simulated blackout conditions). The simulation lab is windowless, and the door does not permit light entry when closed. A manikin (HPS, CAE Healthcare USA, Sarasota, FL, USA) was placed on a standard North Atlantic Treaty Organization (NATO) patient litter in a standard U.S. Air Force patient transport pallet stanchion at a height of 36 in. from the ground. The manikin was placed to approximate the position of a patient undergoing CCATTs transport in the supine position. A second manikin (Ambu Airway Man, Ambu A/S, Ballerup, Denmark) was placed on the ground next to the stanchion to approximate the position of a CCATTs team-transported patient who was floor-loaded, as is often done by forward-deployed teams to facilitate access to the patient, save loading time, and conform to the internal configuration of certain aircraft, such as the Lockheed HC-130P.

Participants attempted intubation of a normal airway with the room lights on (NAL) at the ground level and the stanchion level. Each manikin was capable of simulating a difficult airway scenario using an air bladder located in the manikin’s tongue. The manikins’ airways were altered to simulate a difficult intubation after intubation attempts in both NAL scenarios. The bladders in the manikins’ tongues were inflated with three compressions of a sphygmomanometer bulb for the difficult airway portions of the study to standardize the degree of difficulty. Each participant attempted intubation of the difficult airway under “lights-on” conditions (DAL) at ground and stanchion levels and under “blackout” conditions (DAB) at ground and stanchion levels, where the regular room lights were off and green low-visibility lights were used to minimally illuminate the room, which would occur in an aircraft operating in a combat zone. Each participant repeated the entire sequence for each of the remaining VAIDs. This repetition resulted in the individual participant using each VAID in each of six different scenarios: NAL Ground, NAL Stanchion, DAL Ground, DAL Stanchion, DAB Ground, and DAB Stanchion.

Two additional datasets were collected. First, the amount of time required for each device to be assembled into a ready-to-use state was measured. Assembly was accomplished separately from the intubation scenario and was not included in the “time to intubation” data, but it did include the connection of cables, if any, and the loading or priming of an endotracheal tube so the device was fully prepared for use. This procedure was performed from the disassembled state with the requisite parts of the device placed on a table for the participant to clearly see and under normal room light conditions because CCATT members must inventory and familiarize themselves with their gear upon arrival at their duty station. Second, the time to power on with the device fully assembled was measured. This time frame included the time from the power button or switch being activated to a usable, illuminated image being visible on the device’s screen. The non-parametric Kruskal-Wallis (K-W) test was used to determine if a significant difference was present between the devices.

**Fig. 3:**
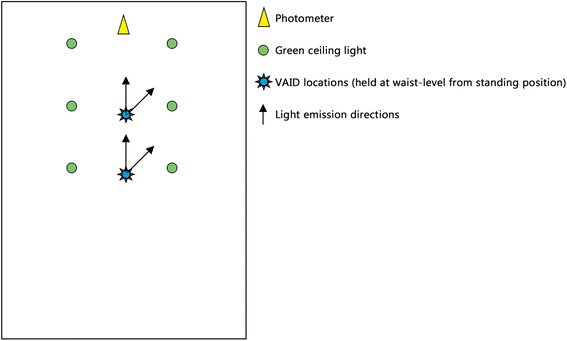
Diagram of the simulation laboratory as used for measuring device light output (scale is approximate)

Second, the amount of light emitted by each VAID was compared. These data were collected independently of the participants’ attempts at intubation and included the U.S. Air Force-issued headlamp for use in low-light conditions on flights (Tactikka with green lens, Petzl, Crolles, France) for comparative purposes. The light emission tests were performed in the CCAT simulation laboratory under the same green, low-light conditions (40-W A-19 Green bulb, Bulbrite, Moonachie, NJ) used during the training simulations, which recreated the lighting conditions in military aircraft operating at night in a combat zone. The light output from the screen of each device and the tip of each device was measured separately (Candella II #C305, Spectra Cine, Burbank, CA, USA). The measurements were taken at distances of 5 and 9 ft, which approximates the distances from the patient to the window of the aircraft in a Lockheed C-130 and Boeing C-17, respectively. Light emission was measured in a direct, head-on fashion and at 45 degrees from direct, and the light analyzer and VAIDs were held between the waist and chest level, which is the position during use on a CCATT’s mission (Fig. 3, Diagram of the simulation laboratory as used for measuring device light output).

Statistical analysis was performed using SAS Version 9.3 (SAS Institute, Cary, NC, USA) and R Version 3.3.3 [12]. Analyses incorporated Dunn’s multi-pairwise comparison tests [13] following K-W test and adjusted *p*-values to control for false discovery rates using the Benjamin-Hochberg (B-H) procedure [14]. Analyses using multi-level mixed effects regression on log-transformed data and nested analysis of variance were performed to account for and assess the impact of the nested study design on the results. Results with a *p*-value of <0.05 were considered statistically significant after adjustments of *p*-values where indicated.

## Results

### Comparison of time to successful intubation

All five VAID’s were compared simultaneously for Novices and Experts to determine whether a significant difference in intubation time existed under each condition (Table 2). All scenarios for Novices reached significance in mean time to intubation, except NAL Stanchion. Three situations reached significance for Experts: DAL Ground, DAB Stanchion and DAB ground. The devices were evaluated as pairs in the scenarios that reached statistical significance (Table 3) to determine where the differences occurred.

**Table 2:** Participants’ meantimes to successful intubation (seconds(SD) [medians])

Condition	Name of devices					K-W *P*-value
	Airtraq	AWS	C-MAC	Coopdech	GlideScope	
Novice group						
NAL Stanchion	14.1 (7.5)[12.5]	15.0 (13.1)[15.0]	17.9 (29.8)[10.4]	14.0 (9.9)[10.9]	17.1 (11.6)[14.1]	0.790
NAL Ground	9.3 (4.6)[9.0]	13.4 (13.5)[9.2]	31.6 (33.0)[16.4]	20.1 (34.7)[11.8]	17.9 (13.6)[13.1]	0.002
DAL Stanchion	13.6 (7.6)[11.9]	10.8 (6.4)[8.9]	23.8 (30.1)[11.2]	19.8 (27.9)[1.1]	22.6 (21.6)[15.1]	0.010
DAL Ground	42.8 (65.3)[14.5]	17.9 (12.4)[16.0]	48.4 (36.3)[39.5]	47.5 (37.4)[35.1]	34.3 (39.4)[22.0]	<0.001
DAB Stanchion	14.3 (7.6)[9.7]	8.5 (5.2)[6.9]	16.9 (11.3)[14.1]	24.3 (58.1)[10.4]	18.4 (16.4)[12.2]	<0.001
DAB Ground	23.0 (48.8)[11.2]	10.8 (6.7)[8.7]	31.0 (27.7)[25.4]	19.0 (18.8)[14.3]	19.8 (10.4)[17.4]	<0.001
Expert group						
NAL stanchion	15.6 (10.5)[13.8]	20.5 (21.0)[13.9]	19.0 (46.3)[9.8]	23.2 (18.8)[15.9]	13.0 (9.1)[10.9]	0.410
NAL ground	10.8 (6.3)[9.6]	12.0 (10.6)[8.4]	12.9 (7.8)[11.1]	14.0 (9.1)[10.8]	9.7 (3.3)[9.7]	0.760
DAL stanchion	11.9 (5.6)[9.8]	12.2 (9.7)[9.8]	18.7 (14.5)[12.6]	14.7 (8.5)[11.5]	15.8 (9.3)[14.0]	0.350
DAL ground	17.6 (7.6)[18.0]	30.1 (93.3)[10.9]	28.5 (21.9)[18.3]	38.0 (37.9)[22.4]	17.2 (5.7)[17.1]	0.011
DAB stanchion	11.3 (6.0)[9.8]	11.6 (13.5)[6.5]	19.0 (22.5)[12.9]	12.5 (5.6)[10.9]	12.4 (5.2)[11.0]	0.033
DAB ground	33.7 (67.7)[10.8]	16.2 (23.8)[11.1]	29.1 (22.9)[21.9]	26.6 (18.4)[17.4]	18.1 (15.8)[12.1]	0.029

*NAL* Normal airway with lights on, *DAL* Difficult airway with lights on, *DAB* Difficult airway with blackout conditions

All values are expressed in seconds (SD). *P* < 0.05 indicated statistical significance

**Table 3:** Paired analysis of devices where the mean time to intubation was statistically significantly different (only pairs where adjusted *p*–values were < 0.05 are shown)

Group		Dunn’s Test *p* value^a^
Novice group		
NAL ground		
	Airtraq^b^ vs. C-MAC	0.002
	Airtraq^b^ vs. GlideScope	0.020
	C-MAC vs. AWS^b^	0.014
DAL stanchion		
	GlideScope vs. AWS^b^	0.050
DAL ground		
	Airtraq^b^ vs. C-MAC	0.027
	Airtraq^b^ vs. Coopdech	0.034
	C-MAC vs. AWS^b^	0.003
	Coopdech vs. AWS^b^	0.006
DAB stanchion		
	C-MAC vs. AWS^b^	0.002
	GlideScope vs. AWS^b^	0.002
DAB ground		
	Airtraq^b^ vs. C-MAC	0.020
	C-MAC vs. AWS^b^	<0.001
	GlideScope vs. AWS^b^	0.004
Expert group		
DAL ground		
	Coopdech vs. AWS^b^	0.009
DAB stanchion		
	C-MAC vs. AWS^b^	0.043
	Coopdech vs. AWS^b^	0.046

*NAL* Normal airway with lights on, *DAL* Difficult airway with lights on, *DAB* Difficult airway with blackout conditions. BH-adjusted *p* < 0.05 indicated statistical significance. ^a^Dunn’s Test *p* value adjusted for false discovery rate using Benjamin-Hochberg (BH) procedure; ^b^The device with shorter time to intubation in the paired results

When compared to other devices in the test in a pair-wise fashion, Novices intubated faster with the AWS and Airtraq, but a direct comparison of AWS and Airtraq revealed no significant differences in any of the scenarios (Table 3).

The DAL Ground scenario and both blackout scenarios (DAB Ground and DAB Stanchion) reached significance in the Expert group, and the devices in these scenarios were compared as pairs. Similar to the Novices, the AWS consistently permitted shorter times to intubation when compared directly to the other devices. However, the Airtraq was less likely to be the faster device (Table 3).

### Comparison of number of optimization maneuvers

The C-MAC required a statistically significant greater number of optimization maneuvers for the Novices to intubate in the DAL Stanchion scenario. Conversely, the AWS in the DAB Ground scenario required a statistically significant fewer number of optimization maneuvers for the Novice group to obtain successful intubation (Table 4). The DAL Stanchion and DAB ground scenarios were further analyzed to determine the odds ratio for each device of the need for an optimization maneuver compared to the AWS (used as a reference because it was the least likely to require one). No odds ratio for any device reached significance versus the AWS in the DAL stanchion scenario. Under DAB Ground conditions, the C-MAC [OR: 6.85 (1.95–24.1), *P* < 0.01] and Coopdech [OR: 4.71 (1.40–15.88), *P* = 0.01] exhibited a significantly higher likelihood of requiring an external manipulation to obtain a satisfactory view for intubation versus the reference AWS.

**Table 4:** Participants’proportion of attempts with one or more optimization maneuvers

Condition	Airtraq	AWS	C-MAC	Coopdech	GlideScope	B-H adjusted *p*-value
Novice group						
NAL stanchion	0.0%	0.0%	4.2%	0.0%	4.2%	0.66
NAL ground	8.3%	4.2%	12.5%	8.3%	8.3%	0.90
DAL stanchion	0.0%	0.0%	25.0%	4.2%	8.3%	0.03
DAL ground	54.2%	33.3%	75.0%	62.5%	58.3%	0.12
DAB stanchion	8.3%	12.5%	29.2%	16.8%	16.8%	0.57
DAB ground	50.0%	29.2%	75.0%	67.7%	45.8%	0.04
Expert group						
NAL Stanchion	0.0%	6.3%	6.3%	0.0%	0.0%	0.55
NAL Ground	6.3%	0.0%	18.8%	12.5%	12.5%	0.55
DAL Stanchion	12.5%	0.0%	18.8%	0.0%	12.5%	0.35
DAL Ground	27.5%	18.8%	75.0%	50.0%	50.0%	0.08
DAB Stanchion	0.0%	0.0%	12.5%	6.3%	18.8%	0.35
DAB Ground	43.8%	12.5%	50.0%	68.8%	25.0%	0.07

*NAL* Normal airway with lights on, *DAL* Difficult airway with lights on, *DAB* Difficult airway with blackout conditions. BH-adjusted *p* < 0.05 indicated statistical significance

A similarly staged analysis was performed for the Expert group. No scenario reached significance when *p*-values were adjusted. However, two scenarios reached significance prior to adjustment: DAL ground and DAB ground (Table 4). The Pentax AWS was chosen as a reference because it was the least likely device to require optimization maneuvers to accomplish intubation. Under DAL ground conditions, the C-MAC (OR: 10.7 (2.08–55.4), *p* < 0.01) was far more likely to require external manipulation. Similarly, the C-MAC (OR: 6.82 (1.16–40.2), *p* = 0.04) and Coopdech (OR: 14.1 (2.33–85.5), *p* = 0.004) exhibited a higher likelihood of requiring optimization maneuvers in DAB ground.

### Subjective ease of use survey

A short survey was performed (https://www.surveymonkey.com) immediately following the conclusion of each participant’s exposure to each device, which allowed participants to rank their perceived ease of intubation for each of the devices (Table 5). The AWS and GlideScope trended toward being perceived as the easiest to use by novices and experts, but statistical significance was not reached in all scenarios. Notably, significance was more likely to be reached in the more difficult scenarios.

**Table 5:** Survey results of participants’ subjective ease of use

Question	BH-adjusted *P*-value	Ranking of devices				
		AWS	GlideScope	C-MAC	Coopdech	Airtraq
Novice group						
Overall ease of use of the device	0.180	4.7	4.8	4.3	3.8	3.7
The ease of use of this device under the following circumstance						
NAL Stanchion	0.130	4.9	4.7	4.9	4.2	4
NAL Ground	0.110	4.6	4.4	4.1	3.5	3.9
DAL Stanchion	0.290	4.6	4.4	4.5	3.8	3.9
DAL Ground	0.001	4.6	3.9	3.2	3.2	3.6
DAB Stanchion	0.130	4.6	4.4	4.4	3.7	2.9
DAB Ground	0.130	4.6	4.6	3.3	3.2	3.2
Expert group						
Overall ease of use of the device	0.400	4.4	4.7	4.6	4.2	3.6
The ease of use of this device under the following circumstance						
NAL stanchion	0.047	4.7	4.7	4.4	4.0	4.6
NAL ground	0.120	4.5	4.7	4.2	3.3	4.4
DAL stanchion	0.120	4.6	4.3	4.0	3.9	4.0
DAL ground	0.088	4.5	3.8	3.4	2.2	4.0
DAB stanchion	0.001	4.4	4.3	3.6	3.9	3.0
DAB ground	0.004	4.5	4.2	3.6	3.3	3.1

*NAL* Normal airway with lights on, *DAL* Difficult airway with lights on, *DAB* Difficult airway with blackout conditions

All values are an average of ratings based on a 5-point Likert scale ranging from “5 = Extremely easy” to “1 = Extremely difficult”. BH-adjusted *P*-value <0.05 indicated statistical significance

### Technical data

Data of novices and experts were pooled in this section. All five devices were compared simultaneously to determine whether differences existed between devices in assembly time, power-on time and the sum total of assembly plus power-on times (Table 6).

**Table 6:** Mean times for assembly and power-on in seconds [seconds (median)]

Variable	Device name					BH-adjusted *p*-value
	Airtraq	AWS	C-MAC	Coopdech	GlideScope	
Assembly time	245.8 (176.8)	69.4 (42.6)	91.2 (18.1)	81.6 (47.9)	44.4 (29.1)	0.012
Power-on time	3.22 (3.3)	4.19 (0.8)	16.58 (0.3)	2.90 (0.8)	2.28 (2.5)	0.0228
Total average time	249 (177.7)	73.59 (41.9)	107.8 (17.9)	84.5 (48)	46.68 (30.9)	0.0228

All values are in seconds (SD). BH-adjusted *p*-value <0.05 indicated statistical significance. Data pooled from Novices and Experts

Assembly time, power-on time and total time individually reached statistical significance. The Airtraq’s assembly time was longer than the other devices, and the C-MAC’s power-on was longer than its competitors. The C-MAC’s total time was closer to the other devices, but the Airtraq remained an outlier.

The greatest measurable light output of all the devices was the device tip of the Airtraq at 5 ft and 0 degrees (0.3 fc). Its light emission at 9 ft and 0 degrees (0.1 fc) was similar to the GlideScope. The light emission of the other VAIDs was negligible at that range. However, the Airtraq’s device emitted less light output at 5 ft and 0 degrees than the issued Tactikka headlight (0.5 fc),which is commonly used by the CCATTs to visualize patients, monitors, and chart materials. Light emission from the screen was negligible for all VAIDs at all of the measurement locations.

## Discussion

These results suggest that the Pentax AWS and GlideScope Ranger are superior to the other devices tested, and both devices are suitable for in-flight use by infrequent and seasoned laryngoscopists. A novice in a critical situation must overcome an experience deficit to promptly and safely address a challenging situation. The patient in need of urgent intubation in the austere environment of the back of an aircraft under combat lighting conditions is indubitably one of these situations. The equipment required to manage the crisis should necessitate minimal assembly and preparation time, allow a high likelihood of rapid success, and should not require excessive additional maneuvers to obtain a satisfactory view of the glottic opening. This equipment permits the novice (or infrequent) and expert laryngoscopist to promptly provide the safest care possible.

The more challenging scenarios (i.e., intubating on the ground as opposed to the more conventional height encountered on a stanchion and blackout conditions compared to normal lighting) were more likely to exhibit a statistically significant difference in the time to intubation. The absolute difference in time to intubation using the various devices was measured in seconds in this study, and an additional 20 to 30 s of severe hypoxemia may be deleterious, especially to a patient who is transported because of a brain injury or myocardial ischemia. The present study was a simulation-based study and not an observation of actual clinical practice. The “fog of war” and awareness of an actual patient suffering injury due to the inability to obtain rapid tracheal intubation may very well accentuate the time differential between an easy-to-use device and a more complex device.

Pentax Medical’s AWS generally required little external manipulation in Novice and Expert groups, and it enabled Novices to rapidly complete intubation. This result was evident because the AWS took the shortest mean amount of time for the Novice users under all four of the difficult airway scenarios and the shortest median amount of time under three of these four scenarios. The AWS has a built-in guide track for the endotracheal tube and a convenient “crosshair” on the screen, which provides a very intuitive feel. These factors may have contributed to the consistently high ratings in the subjective evaluation of ease of use. The Verathon GlideScope Ranger was fastest to boot and assemble, and it seemed to excel in the Expert group when the manikin was positioned on the ground. The blade and the monitor of this device are not rigidly connected, which may facilitate a mechanically advantageous intubating position while maintaining a clear view of the monitor. The GlideScope was not the fastest for intubation, but its short assembly and boot times enabled a quicker theoretical “start-to-finish” summation of time.

Users of the Coopdech required a moderate amount of optimization, and its assembly and startup times were similar to the other devices in the study, with the exception of the C-MAC.

ProdolMeditec’sAirtraq facilitated rapid intubation in the hands of the Experts, but it required a lengthy setup, which is obviously not ideal under urgent, stressful, and austere conditions. The setup was significantly longer than all of the other devices. This study also evaluated assembly under conventional room lighting and calm circumstances rather than blackout emergency conditions. It is certainly plausible that assembly time would increase if performed in the dark and under the stress of knowing that failure to correctly assemble the device may negatively and severely impact the patient’s life. The Airtraq also required a moderate amount of optimization maneuvers to obtain a satisfactory laryngeal view.

Karl Storz Endoskope’s C-MAC was the most structurally similar to conventional direct, Macintosh-bladed laryngoscopes. This device is advantageous for teaching where an instructor can view on the screen exactly what the student is seeing when the student uses the device as a direct laryngoscope, but the similarity to direct laryngoscopy may not make it ideal for use by an infrequent or inexperienced laryngoscopist under austere conditions where seconds count. This similarity may be one reason that its use necessitated the greatest amount of external manipulation to obtain a satisfactory laryngeal view. The time taken to power on was also the longest time of all devices tested. The time to successful intubation with the C-MAC was more comparable to the other VAIDs when used by Expert laryngoscopists.

Notably, when asked to describe the ease of use of each device under a specific scenario, subjects most commonly listed the AWS as easiest to use, but when asked about overall ease of use, Novices and Experts chose the GlideScope as the easiest to use. The Expert group exhibited more variance in which device allowed the most rapid intubation, and a different device was used in the three situations where time to intubate between devices reached statistical significance (DAL Ground, DAB Stanchion, and DAB Ground). Experts easily adapt to the particularities of different devices because of their strong fundamental skill set in airway management.

Light emission is a concern when aircraft operate at night in a combat zone because light may allow a ground-based observer to better localize the aircraft when it takes off or lands and direct fire towards the aircraft. Fortunately, the tips of the devices tested emitted minimal light output when measured directly at distances of 5 and 9 ft and negligible output from their screens at any angle. The devices are generally directed toward the interior of the aircraft and not at the windows, which also minimizes light emission risk. Comparison of the light output of VAIDs to the approved and issued headlamp provided a context for the light emission of VAIDs as a low-risk event.

The present study had several limitations. Assembly time was not evaluated separately for Novices and Experts. This measurement may have been a worthwhile additional investigation or altered the results, but pooled data were used for each of the investigated devices, which lowered the chance of data skewing. Experts have likely used multiple different airway devices throughout their career and may have used one or more of the study devices in the past. Prior experience with a study device was not controlled for in this study. Many of the devices tested are in routine clinical practice across the country, which makes this factor somewhat impractical without significantly increasing the sample size. Light emission was not tested in situ in an actual aircraft by an observer who was external to the aircraft, which may have produced different results.

## Conclusions

The Pentax AWS exhibited the shortest time to successful intubation in all of the difficult airway scenarios in the Novice group, and this device was assembled for use in one of the shortest amounts of time. The GlideScope was also well suited to the intended environment because of its short power-up and assembly times and overall perceived ease of use. The Airtraq exhibited a lengthy setup time, and the C-MAC and Coopdech required frequent external airway manipulations, which make these devices insufficiently adapted to the intended clinical applications for this paper. The Pentax AWS and GlideScope Ranger are suitable for in-flight use by infrequent and seasoned laryngoscopists. None of the devices tested exhibited greater light emission than the standard issue headlamp used by the aircraft crew members, which reduces the chance that light from the device would negatively impact aircraft safety in a combat zone.

## Appendix 1

### AFMESA Market Research Team Capability Requirements^7^

1. Be able to pass Airworthiness Certification (Safe-to-Fly) criteria for all US Air Force Mobility Air Force (MAF) aircraft

2. Be FDA-approved or comply with FDA regulations

3. Provide visualization of the vocal cords and the endotracheal tube passing through the vocal cords to confirm placement of endotracheal tubes

4. Work without requiring hyperextension of the neck while maintaining the patient in C-spine-neutral position with hard cervical collar in place

5. Have power requirements consistent with the limitations of the CCATT allowance

6. Have an anti-fogging capability

7. Should have pediatric capability

8. Support continuous use without recharging or replacing battery for at least 30 min

9. Battery should maintain a full charge in the “Off” position for at least 15 days

10. Device should have a charge indicator

11. Must pass a function test before use

12. Configuration of the device must support incorporation into the CCATT allowance

13. Shall be portable with a carrying case (If not disposable, shall have a ruggedized carrying case)

14. In operational state, device shall have few parts, excluding container and any battery charger or cable

15. Weight must be less than 7 pounds

16. The device must have an availability threshold of 95%

17. Must conform to support requirements of CCATT allowance to be supportable by current Biomedical Equipment Technician (BMET) kit

18. Have an operator’s manual with pre-use/testing procedures and list of any spare/replacement parts

19. Repair of the device must not require use of any proprietary tools or testing equipment

20. Parts should be obtainable by any DoD entity without additional constraints (training, paperwork, etc.)

21. Training must be consistent with other CCATT training and operational tempo

22. Use vendor-provided training material, time to train identified personnel

23. Vendor must provide initial training to clinic staff on functionality

24. Vendor must provide follow-on training to clinic staff on new functionality or enhancements

25. Initial training will take place on-site

26. Shall meet standard infection control guidance as provided by Centers for Disease Control and Prevention (CDC) and US Air Force guidelines

## Abbreviations

AFMESA: Air Force Medical Evaluation Support Activity
CCATT: Critical Care Air Transport Team
CSTARS: Centers for Sustainment of Trauma and Readiness Skills
DAB: Difficult airway- blackout
DAL: Difficult airway- lights on
IRB: Institutional review board
K-W: Kruskal-Wallis
NAL: Normal airway- lights on
NATO: North Atlantic Treaty Organization
UCMC: University of Cincinnati Medical Center
VAID: Video-assisted intubation device
